# Supplementary material for: Analysis of the Clinicopathological Characteristics, Prognosis, and Lymphocyte Infiltration of Esophageal Neuroendocrine Neoplasms: A Surgery-Based Cohort and Propensity-Score Matching Study
Source: Cancers (Basel). 2023 Mar 13;15(6):1732. doi: 10.3390/cancers15061732 (PMC10046526; doi:10.3390/cancers15061732)
Supplement: Supplementary file 1 [file cancers-15-01732-s001.zip › cancers-2072025-supplementary.pdf]

Supplementary Materials

Analysis of the Clinicopathological Characteristics, Prognosis, and Lymphocyte Infiltration of Esophageal Neuroendocrine Neoplasms: A Surgery-Based Cohort and Propensity-Score Matching Study

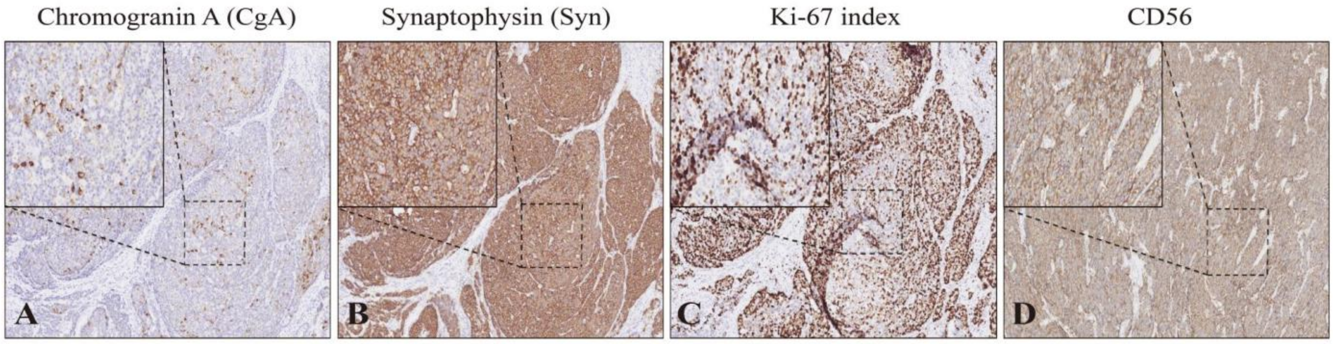

Figure S1. The specific tumor markers for E-NENs under a 400× high-power field. (A. Chromogranin A; B. Synaptophysin; C. CD-56; D. Ki-67 expression).

Table S1. The information of products and reagents related the study.

<table>
<tr><th>Products</th><th>Information</th><th>Remarks</th></tr>
<tr><td>CD4 antibodies</td><td>AB133616, Abcam, Cambridge, UK</td><td rowspan="6">The anti-CD4 and anti-CD8 antibodies were diluted by 3% Bovine Serum Albumin (BSA) solution with a ratio of 1 to 400, while anti-CD20 antibodies' dilution ratio was 1 to 1000</td></tr>
<tr><td>CD8 antibodies</td><td>85336, Cell Signaling Technology, USA</td></tr>
<tr><td>CD20 antibodies</td><td>AB78237, Abcam, Cambridge, UK</td></tr>
<tr><td>Diaminobenzidine chromogen</td><td>CR2204077, Servicebio, CHN</td></tr>
<tr><td>Haematoxylin</td><td>GK500710, GeneTech(Shanghai), CHN</td></tr>
<tr><td>Eosin</td><td>CR2101102, Servicebio, CHN</td></tr>
</table>

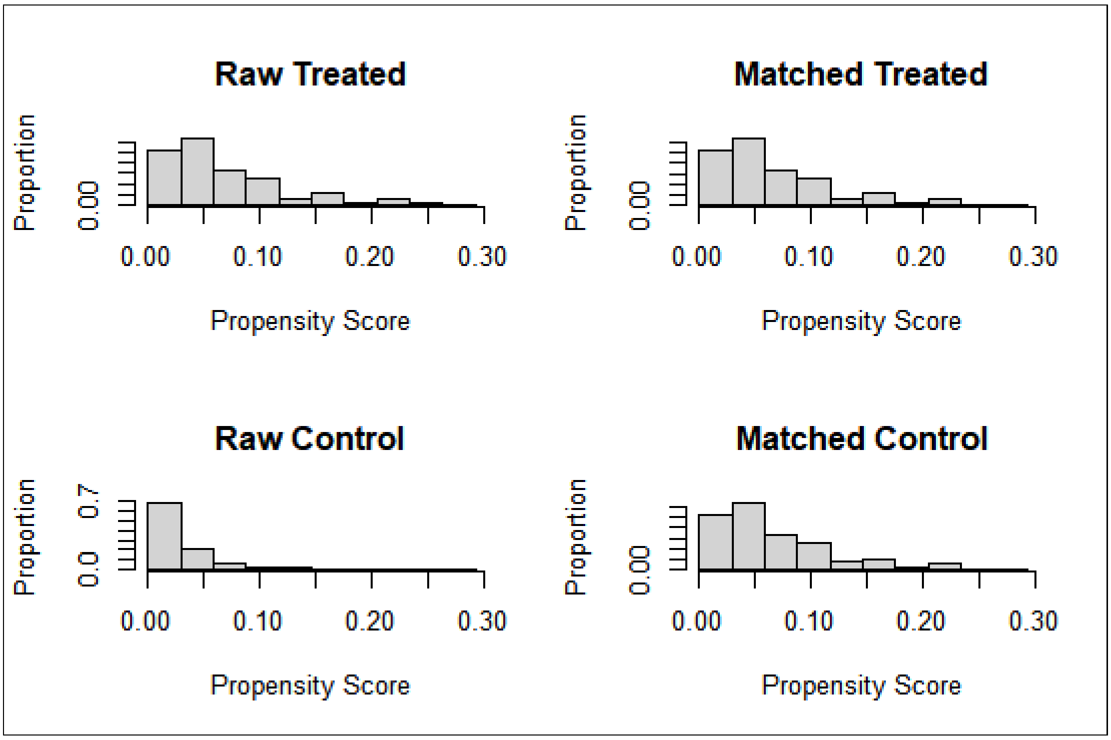

Figure S2. Standardized bias across covariates between ESCC and E-NECs has been well balanced after matching. Variables used for matching included age, tumor location, T status, N status, differentiation, LVI, neoadjuvant and adjuvant therapy. Abbreviations: LVI, lymphovascular invasion; T status and N status were both included pathological category [pT, pN, pStage] and category after neoadjuvant therapy [ypT, ypN, ypStage].

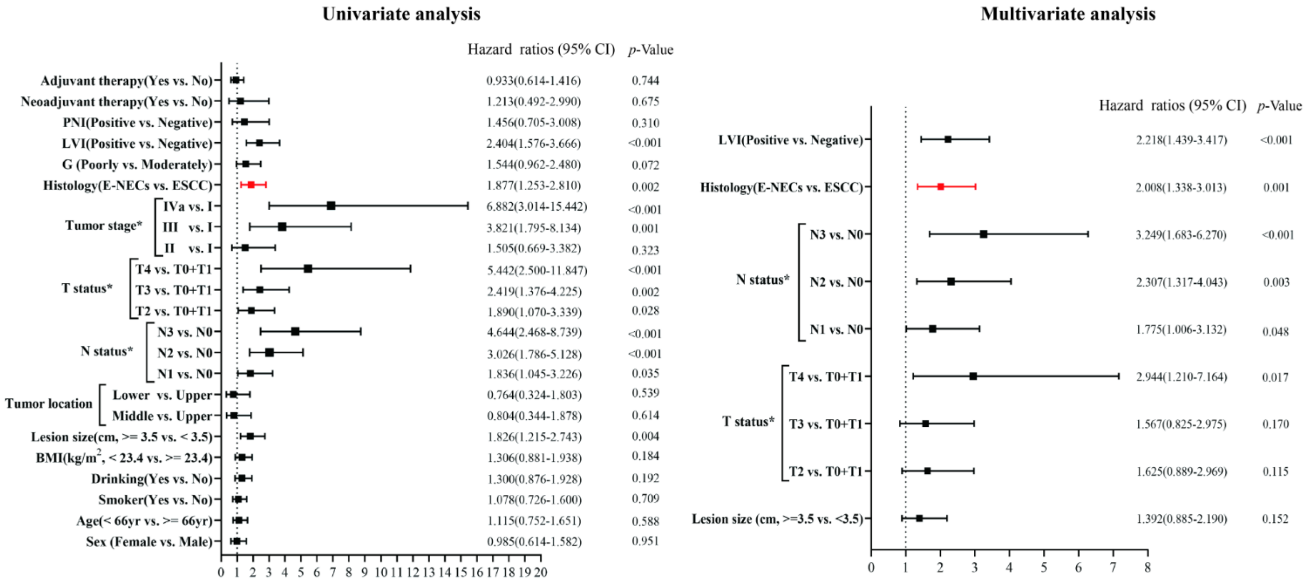

Figure S3. Univariate and multivariate analysis of variables associated with overall survival after matching#. Abbreviations: G, tumor differentiation; ESCC, esophageal squamous cell carcinoma; E-NECs, esophageal neuroendocrine carcinomas; OS, overall survival; BMI, body mass index; LVI, lymphovascular invasion; PNI, perineural invasion. *Both T status, N status, and tumor stage included the pathological category (pT, pN, pStage) and the category (ypT, ypN, ypStage) after neoadjuvant therapy. # The hazard ratios for OS were calculated based on the cohort excluding the 3-cases NET.

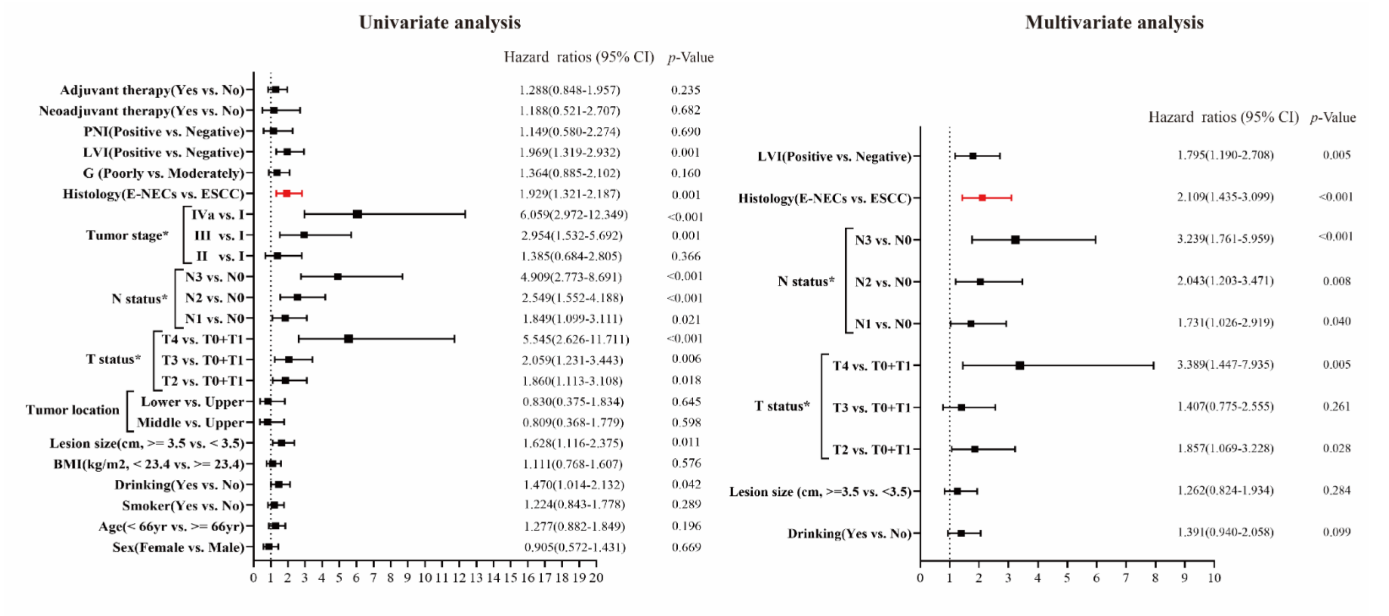

Figure S4. Univariate and multivariate analysis of variables related to recurrence-free survival after matching#. Abbreviations: G, tumor differentiation; ESCC, esophageal squamous cell carcinoma; E-NECs, esophageal neuroendocrine carcinomas; RFS, recurrence-free survival; BMI, body mass index; LVI, lymphovascular invasion; PNI, perineural invasion. *Both T status, N status, and tumor stage included the pathological category (pT, pN, pStage) and the category (ypT, ypN, ypStage) after neoadjuvant therapy. # The hazard ratios for RFS were calculated based on the cohort excluding the 3-cases NET.
